# Accumulated quiescent neural stem cells in adult hippocampus of the mouse model for the *MECP2* duplication syndrome

**DOI:** 10.1038/srep41701

**Published:** 2017-01-31

**Authors:** Zhifang Chen, Xiao Li, Jingjing Zhou, Bo Yuan, Bin Yu, Dali Tong, Cheng Cheng, Yinqi Shao, Shengnan Xia, Ran Zhang, Jingwen Lyu, Xiuya Yu, Chen Dong, Wen-Hao Zhou, Zilong Qiu

**Affiliations:** 1 Institute of Neuroscience, CAS Key Laboratory of Primate Neurobiology, State Kay Laboratory of Neuroscience, CAS Center for Excellence in Brain Science and Intelligence Technology, Shanghai Institutes for Biological Sciences, Chinese Academy of Sciences, 320 Yue-Yang Road, Shanghai, 200031, China; 2 Department of Neonatology, Children’s Hospital of Fudan University, Shanghai, 201102, China

## Abstract

Duplications of Methyl CpG binding protein 2 (*MECP2*) -containing segments lead to the *MECP2* duplication syndrome, in which severe autistic symptoms were identified. Whether adult neurogenesis may play a role in pathogenesis of autism and the role of *MECP2* on state determination of adult neural stem cells (NSCs) remain largely unclear. Using a *MECP2* transgenic (TG) mouse model for the *MECP2* duplication syndrome, we found that adult hippocampal quiescent NSCs were significantly accumulated in TG mice comparing to wild type (WT) mice, the neural progenitor cells (NPCs) were reduced and the neuroblasts were increased in adult hippocampi of *MECP2* TG mice. Interestingly, we found that parvalbumin (PV) positive interneurons were significantly decreased in *MECP2* TG mice, which were critical for determining fates of adult hippocampal NSCs between the quiescence and activation. In summary, we found that MeCP2 plays a critical role in regulating fate determination of adult NSCs. These evidences further suggest that abnormal development of NSCs may play a role in the pathogenesis of the *MECP2* duplication syndrome.

Methyl CpG-binding protein 2 (MeCP2) is a critical transcriptional regulator and also plays an important role in regulating microRNA biogenesis^1^,^2^. Mutations of *MECP2* gene were considered as the major cause of the Rett syndrome, a severe neurodevelopmental disorders^3^. Duplications of genomic segments containing the *MECP2* gene and hence increased MeCP2 protein dosage led to a severe neurological disorder, the *MECP2* duplication syndrome, which is 100% penetrant in affected males and is associated with infantile hypotonia, severe autistic features, poor speech development, recurrent infections, progressive spasticity, and developmental regression^4^,^5^. *MECP2* transgenic mice also exhibit various neurological symptoms, including increased anxiety and fear learning, hypoactive, and early death^6^. Whether adult neurogenesis deficits might be involved in the *MECP2* duplication syndrome remains unclear.

Neural stem cells (NSCs) in the hippocampus generate new neurons throughout adulthood^7^,^8^. Most of the adult NSCs are relatively quiescent. They will be activated or maintain quiescent in response to extrinsic stimuli or intrinsic signals^9^,^10^. Dysregulation of NSCs may contribute to pathogenesis of various neuropsychiatric disorders such as depression and schizophrenia. Previous work identified local parvalbumin (PV) positive interneurons play a critical role in controlling quiescent state of adult NSCs^11^. Interestingly, numbers of PV positive interneurons were found to increase in *Mecp2* null mice^12^. However, whether MeCP2 may have direct influence on fate determination of adult NSCs remains unknown.

In this work, we showed that adult hippocampal quiescent NSCs were accumulated, the neural progenitor cells (NPCs) were reduced and the neuroblasts were increased in hippocampus of *MECP2* TG mice. Furthermore, we found that PV positive interneurons, which were identified as a critical niche component that dictates the adult hippocampal NSCs between the quiescence and activation^11^, were significantly decreased in dentate gyrus (DG) of *MECP2* TG mice. These findings directly link MeCP2 to fate determination of adult NSCs in hippocampus and further suggest that adult neurogenesis may play a critical role in pathogenesis of autism spectrum disorders.

## Results

### Overexpression of MeCP2 expands the adult NSC pool *in vivo*

**Figure 1:**
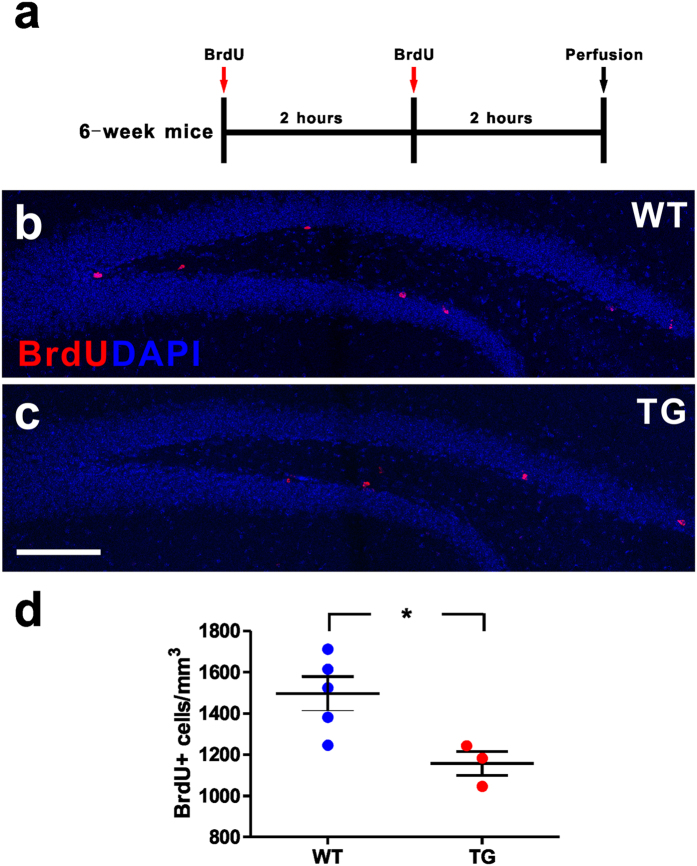
Reduced adult hippocampal dividing cells in *MECP2* transgenic mice. **(a)** Experimental scheme for assessing new dividing cells in the adult hippocampus. **(b**,**c)** Confocal microscopy images of the adult hippocampus showing dividing cells in the subgranular zone (SGZ) of wild type (WT) and *MECP2* transgenic (TG) mice, displaying BrdU staining (red). The nuclear label DAPI is shown in blue. Scale bar: 200 μm. **(d**) Quantitative analysis of BrdU label density in granule cell layer. Values are Mean ± S.E.M (n = 5 WT; n = 3 TG; *P < 0.05, student’s t-test).

To determine whether MeCP2 overexpression has an impact on adult hippocampal neurogenesis *in vivo*, we first assessed the dividing cells of hippocampus in both WT and *MECP2* TG mice. The dividing cells were identified by the incorporation of BrdU administered through intraperitoneal injections into adult mice twice with a two-hour interval, and analyzed at two hours after the last BrdU injections (Fig. 1a). Quantitative histological ananlysis showed that *MECP2* TG mice had fewer BrdU^+^ cells in the DG, compared with WT mice (Fig. 1b–d). There are at least two types of proliferating cells that can be labeled by BrdU, such as GFAP^+^/Nestin^+^ radial glia-like NSCs (RGLs) and GFAP^−^/Nestin^+^ NPCs^13^.

**Figure 2:**
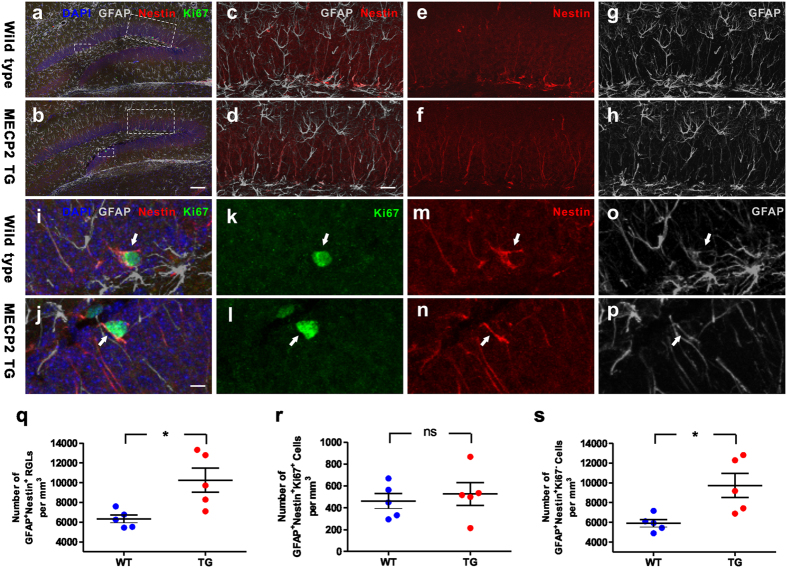
Increased adult quiescent NSCs in the hippocampal dentate gyrus of *MECP2* transgenic mice. **(a**,**b)** Sample confocal images of immunostaining of DAPI, GFAP, Nestin and Ki67. **(c–h)** High-magnification images of the large boxes in picture A and B showed the adult NSCs (GFAP^+^/Nestin^+^) in wild type mice and *MECP2* transgenic (TG) mice. **(i–p)** High-magnification images of the small boxes in picture A and B showed the proliferating RGLs (GFAP^+^/Nestin^+^/Ki67^+^; arrows) in wild type mice and *MECP2* transgenic mice. Scale bar: (**a**,**b**) 100 μm; (**c**–**h**) 25 μm; (**i**–**p**) 5 μm. **(q–s)** Quantitative analysis of GFAP^+^/Nestin^+^/RGLs, GFAP^+^/Nestin^+^/Ki67^+^ proliferating RGLs, and GFAP^+^/Nestin^+^/Ki67^−^ quiescent RGLs density in granule cell layer respectively. Values are Mean ± S.E.M (n = 5 animals for each genotype; *P < 0.05, student’s t-test; ns: non-significant).

To distinguish which type of cells were affected in *MECP2* TG mice, we stained the brain sections with antibodies against GFAP, Nestin and ki67. We found that RGLs (GFAP^+^/Nestin^+^) were increased in DG of TG mice, in comparison to WT mice (Fig. 2a–h,q), and proliferating RGLs (GFAP^+^/Nestin^+^/Ki67^+^) appeared not to be different between WT and *MECP2* TG mice (Fig. 2i–p,r). Consequently, adult quiescent RGLs (GFAP^+^/Nestin^+^/Ki67^−^) were significantly increased in *MECP2* TG mice (Fig. 2s). These data suggest that the adult NSC pool is expanded in hippocampi of *MECP2* TG mice.

### The neural progenitor cells are reduced and the neuroblasts are increased in *MECP2* transgenic mice

**Figure 3:**
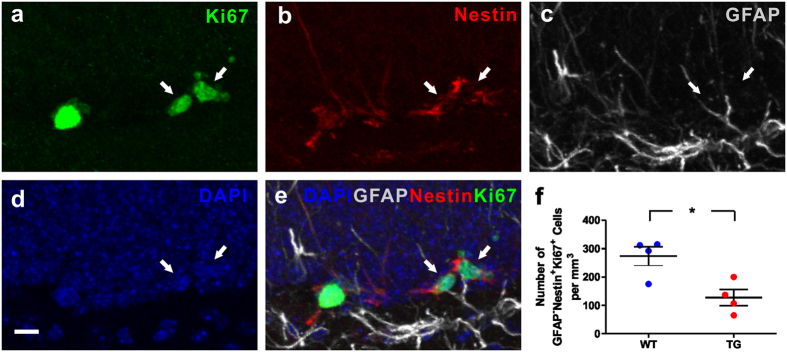
Decreased adult neural progenitor cells in the hippocampal dentate gyrus of *MECP2* transgenic mice. **(a–e**) Sample confocal images of immunostaining of DAPI, GFAP, Nestin and Ki67. Arrows indicate GFAP^−^/Nestin^+^/Ki67^+^ progenitor cells. Scale bar: 10 μm. **(f)** Quantitative analysis of GFAP^−^/Nestin^+^/Ki67^+^ progenitor cells density in granule cell layer. Values are Mean ± S.E.M (n = 4 animals for each genotype; *P < 0.05, student’s t-test).

To further characterize the fewer dividing cells in DG of *MECP2* TG mice, we examined the NPCs in WT and TG mice. We found that the NPCs (GFAP^−^/Nestin^+^/Ki67^+^) were reduced in DG of TG mice, in comparison to WT mice (Fig. 3). These results suggest that the differentiation of RGL into NPCs or the proliferation of NPCs might be hampered in adult *MECP2* TG mice.

**Figure 4:**
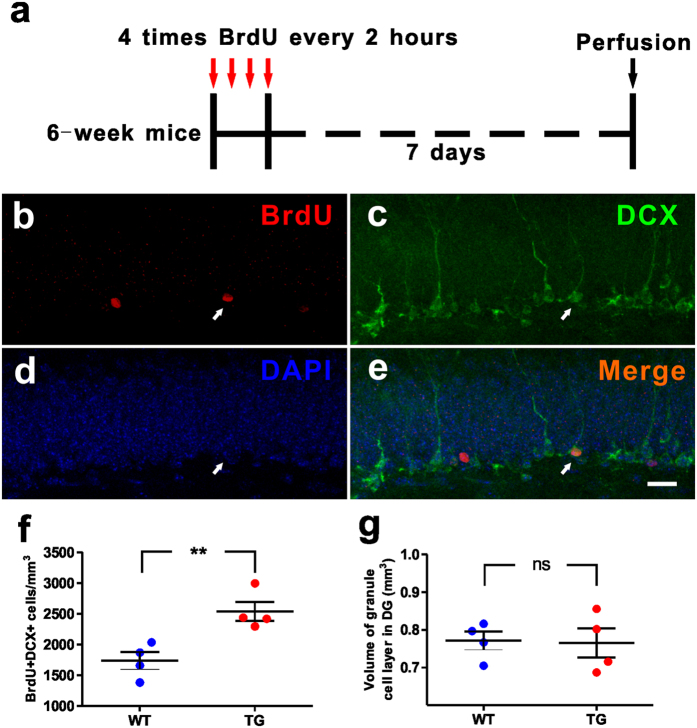
Increased neuroblasts in the hippocampal dentate gyrus of *MECP2* transgenic mice. **(a)** Experimental scheme for assessing new neuroblasts in the adult hippocampus. **(b–e)** Sample confocal images of immunostaining of DAPI, MCM2 and DCX. Arrows indicate BrdU^+^/DCX^+^ neuroblasts. Scale bar: (**b**–**e**), 20 μm. (**f**) The dentate gyrus of *MECP2* TG mice exhibited increased BrdU^+^/DCX^+^ neuroblasts analyzed at 7 days after 4 doses of BrdU injections. **(g**) *MECP2* TG mice had similar DG volume with WT mice. Values are Mean ± S.E.M (n = 4 animals for each genotype; **P < 0.01, ns: non-significant, student’s t-test).

To identify whether neuronal differentiation is affected in the adult hippocampus of TG mice, we gave four doses of BrdU injections with two-hour intervals to label the dividing cells in the DG and analyzed the proliferating neuroblasts at 7 days after the last BrdU injection (Fig. 4a). We counted neuroblasts (BrdU^+^/DCX^+^) and found that the neuroblasts were increased in DG of TG mice, in comparison to WT mice (Fig. 4b–f). In addition, WT and *MECP2* TG mice exhibited similar volume of granule cell layer in DG (Fig. 4g). These results suggest that MeCP2 overexpression may play an important role in the neuronal differentiation of NPCs into neuroblasts or the proliferation of neuroblasts in adult *MECP2* TG mice.

### MeCP2 overexpression has no impacts on proliferation or differentiation of adult NPCs *in vitro*

**Figure 5:**
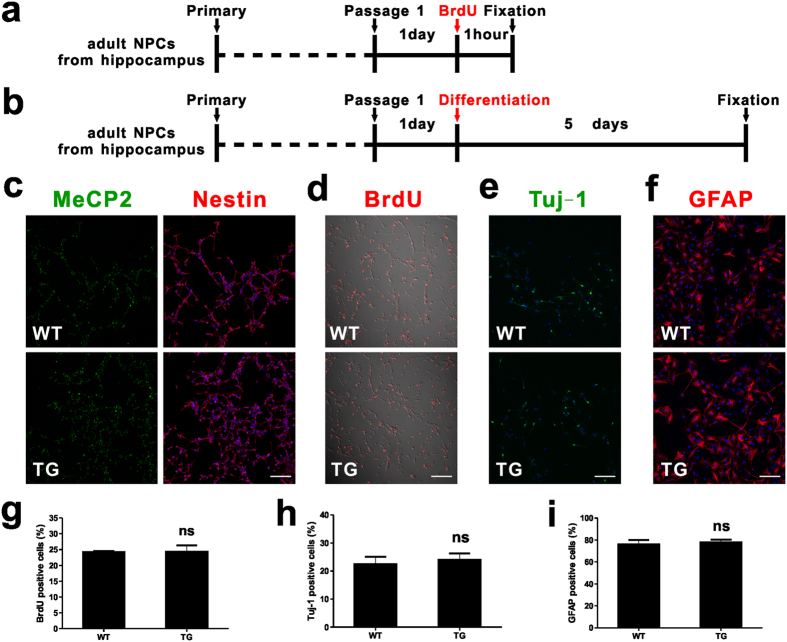
Proliferation and differentiation of adult hippocampal NPCs *in vitro.* (**a**) Experimental scheme for assessing proliferation of NPCs *in vitro*. **(b)** Experimental scheme for assessing differentiation of NPCs *in vitro*. **(c)** Adult hippocampal NPCs culture under proliferating conditions expressed the neural progenitor cell marker Nestin (cytoplasmic, red); MeCP2 in green; Dapi in blue. Scale bar: 100 μm. **(d)** Both WT and TG NSCs incorporate the thymidine analog, BrdU, under proliferating conditions (BrdU, red). Scale bar: 100 μm. **(e)** Differentiation of adult NSCs into neurons (Tuj-1, green) by 1 μM RA and 5 μM forskolin. Scale bar: 100 μm. **(f)** Differentiation of adult NSCs into astrocytes (GFAP, red) by 1% FBS. Scale bar: 100 μm. **(g**–**i)** Quantitative analysis showing the percentages of BrdU^+^, Tuj-1^+^, and GFAP^+^ cells in WT and TG individuals respectively. Values are Mean ± S.E.M (ns: non-significant, student’s t-test).

To elucidate the functions of MeCP2 overexpression in adult NPCs, we isolated adult NPCs from the DG of *MECP2* TG mice and WT littermates. We found that nearly all cultured cells were positive for the progenitor marker Nestin (Fig. 5c), and MeCP2 was expressed in both WT and TG NPCs (Fig. 5c). We pulsed the cells with BrdU for one hour to assess the proliferation of these NPCs (Fig. 5a) and found that NPCs from *MECP2* TG mice exhibited as much BrdU incorporation as WT NPCs (Fig. 5d,g). To assess the effect of MeCP2 overexpression on adult NSC differentiation, both WT and *MECP2* TG NPCs were differentiated for five days (Fig. 5b), differentiated cells were stained with Tuj-1 (Fig. 5e) for neurons and GFAP for astrocytes (Fig. 5f). We found that adult NPCs from *MECP2* TG mice exhibited similar results in neuronal and astrocytic differentiation compared with adult NPCs from WT mice (Fig. 5h,i). These results suggest that MeCP2 overexpression do not alter the proliferation and differentiation of adult NPCs *in vitro*. These *in vitro* results along with *in vivio* data indicate that there may be non-cell autonomous influence of niche cells on fate decision of adult NPCs in *MECP2* TG mice.

### MeCP2 regulates parvalbumin interneurons involved in neurogenesis

**Figure 6:**
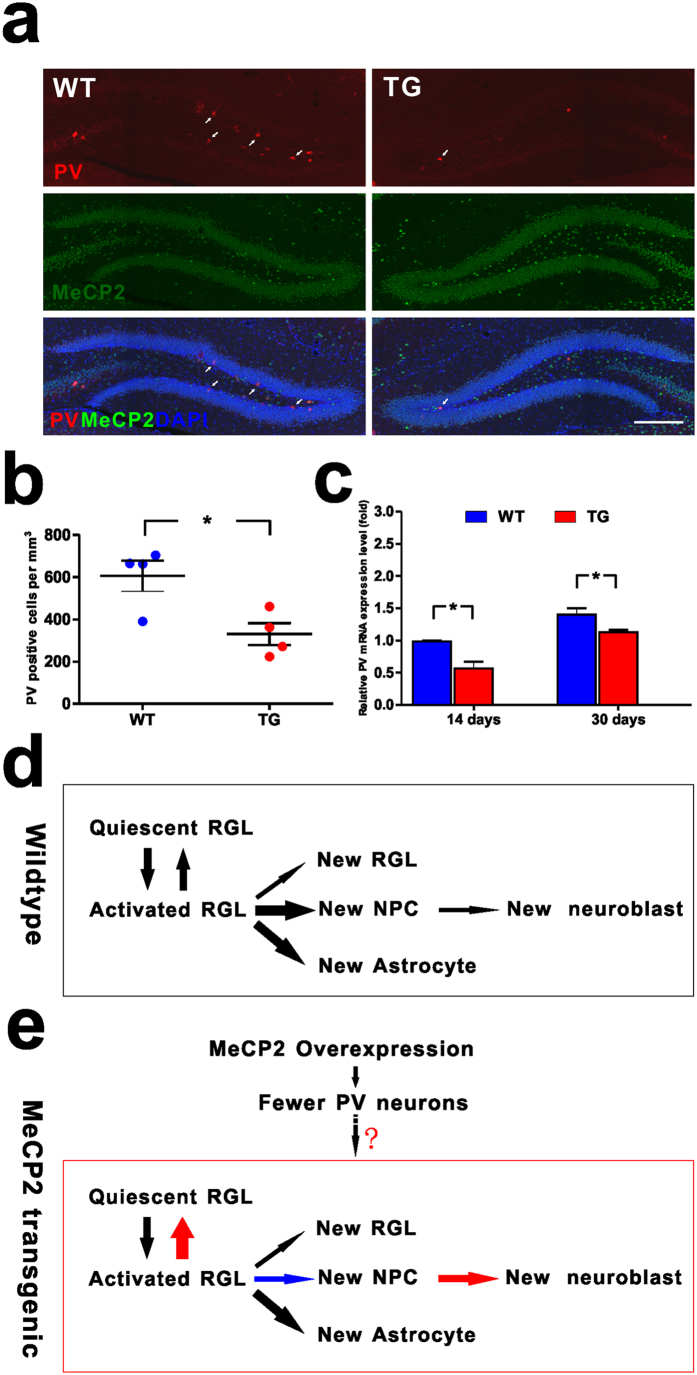
MeCP2 regulates parvalbumin interneurons involved in adult neurogenesis. **(a)** Density of parvalbumin interneurons in the DG significantly decreased in *MECP2* transgenic mice. Representative images showing the parvalbumin interneurons (red, arrows) in DG were along with the border of the granule cell layer (MeCP2 in green, Dapi in blue). Scale bar: 200 μm. **(b)** Quantitative analysis of parvalbumin interneurons density in granule cell layer. Values are Mean ± S.E.M (n = 4 animals for each genotype; *P < 0.05, student’s t-test). (**c)** Expression of PV mRNA decreased in *MECP2* transgenic mice (n = 3 animals for each genotype; *P < 0.05, student’s t-test). **(d)** A model of RGLs/NPCs behaviors in the adult WT mouse hippocampus. an activated RGL usually have four choice points: (1) to be quiescent; (2) symmetric self-renewal to expand the RGL pool; (3) neurogenic asymmetric self-renewal to generate a NPC; (4) astrogliogenic asymmetric self-renewal to generate an astrocyte (modified from Bonaguidi, 2011). The thickness of the arrow indicates the relative probability of each choice. **(e)** A model on the role of MeCP2 overexpression in regulating RGLs/NPCs in the adult MECP2 transgenic mouse hippocampus. MeCP2 overexpression promotes activated RGLs return to quiescence; and inhibits neurogenic asymmetric self-renewal to generate a NPC or the proliferation of NPCs; also, MeCP2 overexpression promotes the differentiation of NPCs into neuroblasts or the proliferation of neuroblasts. MeCP2 overexpression reduced parvalbumin interneurons. Low activity of parvalbumin interneurons as a result of *MeCP2* overexpression could be responsible for the altered neurogenesis in *MECP2* TG mice. Red and blue arrows indicate increased and decreased probability, respectively.

Pavalbumin (PV) interneurons were identified as a critical niche component for adult hippocampal neurogenesis, and was shown to suppress adult quiescent NSCs activation in the adult DG^11^. To examine the expression of PV in *MECP2* TG mice, we stained the brain sections with antibody against PV and found that the density of PV^+^ neurons was significantly decreased in DG of *MECP2* TG mice compared with WT mice (Fig. 6a,b). Consistently, mRNA expression of PV decreased in *MECP2* TG mice at different stages before adulthood compared with WT mice (Fig. 6c). These results indicate that decreased expression of PV as a result of MeCP2 overexpression could be responsible for the altered neurogenesis in *MECP2* TG mice.

## Discussion

Previous study showed there was no significant difference in cell proliferation and neuronal differentiation between *Mecp2* KO and WT NSCs *in vitro*^14^. Moreover, *Mecp2* KO mice exhibited no deficits in the number of BrdU^+^ cells at either 1 day post-BrdU injection or 4 weeks post-BrdU injection^14^. BrdU^+^ cells in *Mecp2* KO mice differentiated into similar numbers of new neurons compared to WT mice. It was suggested that *Mecp2* knockout had no impact on proliferation and differentiation of adult hippocampus NSCs *in vivo* and *in vitro*^14^. Another research showed ectopically expressed MeCP2 in cultured NPCs was able to enhance neuronal differentiation and suppress astrocytic differentiation compared to the control after virus infection, suggesting a possible function of MeCP2 overexpression in NSCs differentiation^15^. Interestingly, there is no reported effect of MeCP2 overexpression on adult NSCs *in vivo*. We now provide the first study showing the function of MeCP2 overexpression in the quiescence and activation of NSCs in the adult brain of *MECP2* transgenic mice. In the present study, we have uncovered a role for MeCP2 overexpression in the accumulation of adult quiescent NSCs, decreased NPCs and increased neuroblasts in the adult hippocampus, further suggesting the potential involvement of adult neurogenesis in *MECP2*-related autism spectrum disorders.

Since we found no differences in proliferation and differentiation between MeCP2 overexpression and wildtype adult NPCs *in vitro,* we considered that there is non-cell autonomous influence of MeCP2 overexpression niche cells on adult NPCs fate decision in *MECP2* TG mice. Adult neurogenesis arises from neural stem cells within specialized niches. The local niche is associated with regulating multiple stages of adult neurogenesis, from neural progenitor proliferation to new neuron maturation, synaptic integration and survival^16^. It was identified that PV interneurons, one of GABA-releasing niche cells among multiple interneuron subtypes in adult DG, interact functionally with adult NSCs^11^. A study showed that relative PV mRNA expression level was significantly increased in the primary visual cortex of adult *Mecp2* KO mice as determined by RT-qPCR^12^. Another recent study showed decreased number of parvalbumin-expressing interneurons in the medial prefrontal cortex in autism^17^. We detected significant decrease in the number of PV interneurons in DG of *MECP2* TG mice. MeCP2 overexpression may not affect the total number of cells in DG of adult hippocampus, but it can contribute to the decreased parvalbuming interneurons linked to altered neurogenesis^11^,^18^.

Our data showed that in *MECP2* TG mice, adult hippocampal quiescent NSCs were significantly accumulated. NPCs were reduced and neuroblasts were increased. We also found that parvalbumin positive interneurons were significantly decreased in *MECP2* TG mice, which were critical for determining fates of adult hippocampal NSCs between the quiescence and activation. These evidences further suggest that abnormal development of NSCs may play a role in the pathogenesis of the *MECP2* duplication syndrome. Low activity of parvalbumin interneurons as a result of MeCP2 overexpression could be responsible for the altered neurogenesis in *MECP2* TG mice (Fig. 6d,e).

## Methods

All experiments were performed in accordance with the guidelines and under the approval of the Animal Care and Use Committee of the Shanghai Institute for Biological Science of the Chinese Academy of Sciences.

### Animals

The *MECP2* transgenic (TG) line on FVB background was generated from Dr. Huda Zoghbi laboratory and purchased from the Jackson Laboratory (#008679)^6^. Heterozygous male was crossed with WT female to maintain a live colony.

### BrdU incorporation

For *in vivo* analysis of adult NSCs, adult (6 weeks old) male mice were injected with BrdU twice (50 mg/kg in saline, every 2 hours, Sigma, flu/Ald, B5002) intraperitoneally, and then were sacrificed at 2 hours post-last injection.

For *in vivo* analysis of newborn neurons, adult (6 weeks old) male mice were injected with BrdU for 4 times (50 mg/kg in saline, every 2 hours, Sigma, flu/Ald, B5002) intraperitoneally, and then were sacrificed at 7 days post-last injection.

For *in vitro* analysis of adult NSCs proliferation, BrdU was incorporated into the medium at a concentration of 100 μg/ml for one hour at 37 °C.

### Adult NSCs culture, proliferation and differentiation assay

Adult NSCs were prepared as described previously^19^. For proliferation assay, neurospheres were dissociated into single cells, and seeded onto coverslips at a density of 50,000/ml. The next day, BrdU was incorporated into the medium at a concentration of 100 μg/ml for one hour at 37 °C. Coverslips were fixed with 4% PFA for 10 min at room temperature, washed three times for 5 min each in 0.01 M PBS. For differentiation assay, neurospheres were dissociated into single cells, and seeded onto coverslips at a density of 100,000/ml. The next day, the medium was replaced with 1 μM RA (Sigma) and 5 μM forskolin (Sigma) (differentiated into neuron) or 1% FBS (differentiate into astrocyte). Five days later, coverslips were fixed with 4% PFA for 10 min at room temperature, washed three times for 5 min each in 0.01 M PBS and were further performed immunostaining work.

### Tissue preparation and Immunostaining

Mice were euthanized by intraperitoneal injection of sodium pentobarbital, and then transcardially perfused with saline followed by 4% paraformaldehyde (PFA). Brains were dissected out, post-fixed overnight in 4% PFA, and then dehydrated in 30% sucrose.

Coronal sections of 40-μm thickness were cut on the freezing microtome (LEICA CM1950) and stored in PBS.

For Brdu immunostaining, free-floating sections were incubated in 2 N HCl for 15 min at 37 degree then neutralized in 0.1 M boric acid solution (pH 8.5) for 10 minutes and washed by PBS for 3 times (each time for 5 minutes). Primary antibodies used were as follow: Mouse BrdU (1/5000, Millipore), Chicken GFAP (1/1000, Chemicon), Mouse Nestin (1/800, R&D), Rabbit Ki67 (1/400, Abcam), Rabbit MCM2 (1/500, Abcam), Goat DCX (1/200, Santa Cruz), Rabbit MeCP2 (1/1000, Cell signaling), Rabbit Tuj-1 (1/200, Abcam), Mouse PV (1/1000, Millipore). Fluorescently conjugated secondary antibodies (Invitrogen; 1/1000) were used. Nuclei were stained with 4′,6-Diamidino-2-phenylindole (DAPI; Sigma).

### Confocal imaging and cell quantification

Confocal z-stack images were acquired on a Nikon A1 confocal laser microscope system.

For *in vivo* experiments, NIH Image J with NeuronJ plugin was used to count the immunoreactive cell number at the subgranular zone (SGZ) in the dentate gyrus and to quantify the area of granule cell layer in the dentate gyrus. GFAP^+^ Nestin^+^ indicates RGL, GFAP^+^ Nestin^+^ Ki67^+^ indicates proliferating RGL, GFAP^+^ Nestin^+^ Ki67^−^ indicates quiescent RGL, GFAP^−^Nestin^+^ Ki67^+^ indicates neural progenitor cell, and BrdU^+^ DCX^+^ indicates neuroblast. The density of cells was determined by dividing the total number of immunoreactive cells by the corresponding volume (the area * 40 μm) of granule cell layer.

For the analysis of *in vitro* proliferating adult NSCs or differentiated neurons or astrocytes, Image J was used to count the immuoreactive cell numbers. The percentage of immunoreactive cells was determined by dividing the number of immunoreactive cells by the total number of cells. A minimum of 1000 cells were analyzed for each culture condition.

### Quantitative real time PCR

Total RNA was collected from fresh hippocampus dentate gyrus tissue for reverse transcription (Bio-Rad, 1708891). 2× SYBR Green Master Mix (TOYOBO, QPK201) AND QIAGEN Rotor-Gene Q machine were used in real time PCR experiments. Mouse PV real time primers were used as before^20^.

### Statistical analysis

The results were expressed as Mean ± S.E.M. The two-tailed Student’s t-test was carried out for the analysis of animal-related *in vivo* and *in vitro* results. Significance was set as follows: *P < 0.05, **P < 0.01.

## Additional Information

**How to cite this article:** Chen, Z. *et al*. Accumulated quiescent neural stem cells in adult hippocampus of the mouse model for the *MECP2* duplication syndrome. *Sci. Rep.* **7**, 41701; doi: 10.1038/srep41701 (2017).

**Publisher's note:** Springer Nature remains neutral with regard to jurisdictional claims in published maps and institutional affiliations.
